# Complete Genetic Analysis of Plasmids Carrying *mcr-1* and Other Resistance Genes in Avian Pathogenic Escherichia coli Isolates from Diseased Chickens in Anhui Province in China

**DOI:** 10.1128/mSphere.01135-20

**Published:** 2021-04-14

**Authors:** Dongdong Yin, Baoyan Cheng, Kankan Yang, Mei Xue, Yanfeng Lin, Zhonghong Li, Xiangjun Song, Ying Shao, Jian Tu, Peng Li, Kezong Qi

**Affiliations:** a Anhui Province Key Laboratory of Veterinary Pathobiology and Disease Control College of Animal Science and Technology, Anhui Agricultural University, Hefei, China; b Chinese PLA Center for Disease Control and Prevention, Beijing, China; c College of Environmental and Chemical Engineering, Nanchang Hangkong University, Nanchang, China; Escola Paulista de Medicina/Universidade Federal de São Paulo

**Keywords:** APEC, colistin resistance, IncI2, *mcr-1*, plasmids

## Abstract

Antimicrobial resistance associated with colistin has emerged as a significant concern worldwide, threatening the use of one of the most important antimicrobials for treating human disease. This study aimed to investigate the prevalence of colistin-resistant avian-pathogenic Escherichia coli (APEC) and shed light on the possibility of transmission of *mcr-1* (mobilized colistin resistance)-positive APEC. A total of 72 APEC isolates from Anhui Province in China were collected between March 2017 and December 2018 and screened for the *mcr-1* gene. Antimicrobial susceptibility testing was performed using the broth dilution method. Pulsed-field gel electrophoresis, Southern blot analysis, and conjugation assay were performed to determine the location and conjugative ability of the *mcr-1* gene. Whole-genome sequencing and analysis were performed using Illumina MiSeq and Nanopore MinION platforms. Three APEC isolates (AH25, AH62, and AH65) were found to be positive for the *mcr-1* gene and showed multidrug resistance. The *mcr-1* genes were located on IncI2 plasmids, and conjugation assays revealed that these plasmids were transferrable. Notably, strains AH62 and AH65, both belonging to ST1788, were collected from different places but carried the same drug resistance genes and shared highly similar plasmids. This study highlights the potential for a possible epidemic of *mcr-1*-positive APEC and the urgent need for continuous active monitoring.

**IMPORTANCE** In this study, three plasmids carrying *mcr-1* were isolated and characterized from APEC isolates from Anhui Province in China. The *mcr-1* genes were located on IncI2 plasmids, and these plasmids were transferrable. These three IncI2 plasmids had high homology with the plasmids harbored by pathogenic bacteria isolated from other species. This finding showed that IncI2 plasmids poses a risk for the exchange of genetic material between different niches. Although colistin has been banned for use in food-producing animals in China, the coexistence of the broad-spectrum β-lactamase and *mcr-1* genes on a plasmid can also lead to the stable existence of *mcr-1* genes. The findings illustrated the need to improve the monitoring of drug resistance in poultry systems so as to curb the transmission or persistence of multidrug-resistant bacteria.

## INTRODUCTION

Colistin is considered the last defense against multidrug-resistant (MDR) Gram-negative bacteria, and the World Health Organization has classified it as one of the most critical antibiotics (1). Before 2016, colistin was generally used as a feed additive in farms to prevent diseases caused by members of the *Enterobacteriaceae*. This resulted in a significant increase in the rate of resistance to colistin among organisms isolated from livestock and poultry farms, threatening public health (2).

The mobilized colistin resistance gene (*mcr-1*) is a transferable phosphoethanolamine transferase-encoding gene that can modify the lipopolysaccharide of the outer membrane of bacteria, resulting in a weakened binding of colistin to the outer membrane and resistance to the drug (3, 4). The *mcr-1* gene was first identified in 2016 and has been found in more than 50 countries on six continents, indicating that *mcr-1* has become an epidemic worldwide (5–7).

Numerous retrospective studies have shown that chickens are a reservoir of resistance genes, and it is important to identify the origins of their MDR plasmids (8–10). The present study aimed to characterize three avian-pathogenic Escherichia coli (APEC) isolates by Illumina short-read and MinION long-read whole-genome sequencing (WGS) and identify the genetic features of plasmids containing the *mcr-1* genes. A total of 72 APEC isolates were collected from diseased chickens in Anhui Province in China between March 2017 and December 2018 and screened for the *mcr-1* gene.

## RESULTS

### Antimicrobial susceptibility testing.

**FIG 1:**
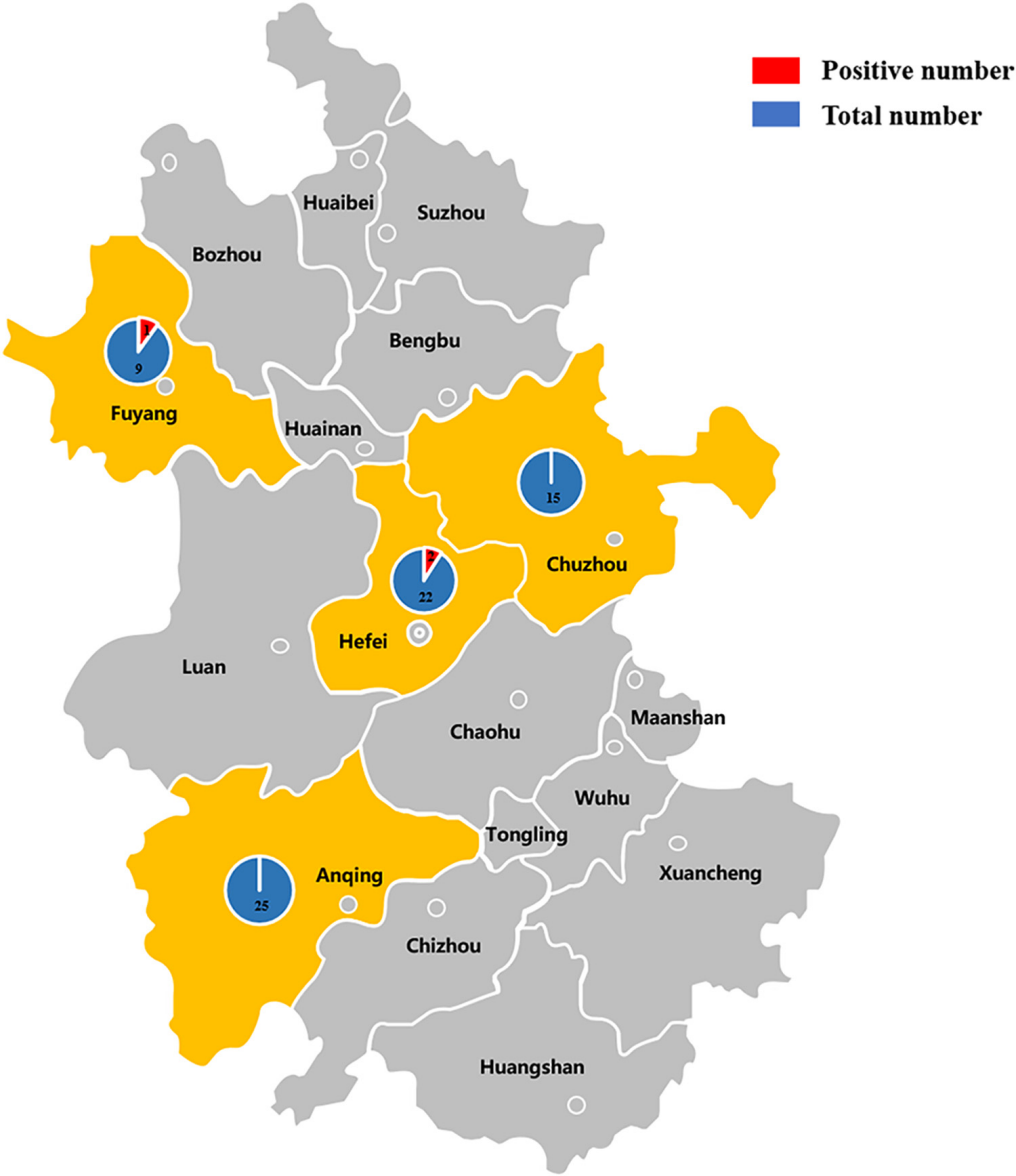
Anhui Province (in gray) and cities (in yellow) where APEC-infected chickens were collected in this study.

Three (∼4%) colistin-resistant isolates (AH25, AH62, and AH65) were identified from a total of 72 APEC strains from Anhui Province during 2017-2018 (Fig. 1). AH25 and AH62 were collected from two different farms in Hefei, while AH65 was collected from a farm in Fuyang. Susceptibility testing indicated that AH25 coexpressed cefotaxime and colistin resistance and exhibited an MDR phenotype, which included resistance to kanamycin, fosfomycin, florfenicol, tetracycline, and polymyxin (see Table S1 in the supplemental material). Both AH62 and AH65 were susceptible to fosfomycin and cefotaxime. The MIC of colistin for the donor strains and their transconjugants was 8 μg/ml (Table S1).

10.1128/mSphere.01135-20.3 TABLE S1 Antibiotic susceptibilities of the three APEC strains and their transconjugants with plasmids harboring *mcr-1*. Download Table S1, DOCX file, 0.03 MB. Copyright © 2021 Yin et al. 2021 Yin et al. https://creativecommons.org/licenses/by/4.0/ This content is distributed under the terms of the Creative Commons Attribution 4.0 International license.

### Microbiological and genomic features of *mcr-1*-positive APEC strains.

All three isolates were subtyped by multilocus sequence typing (MLST), and the corresponding serotypes were identified *in silico* following analysis of the WGS data. AH25 belonged to sequence type 156 (ST156) (serotype O125ab:H28), and AH62 and AH65 belonged to ST1788 (O15:H40) (Table 1). S1 nuclease–pulsed-field gel electrophoresis (S1-PFGE) showed that AH25 contained two different plasmids and that both AH62 and AH65 contained three plasmids (Fig. S1). Southern blot analysis revealed that the *mcr-1* genes were located on an ∼64-kb plasmid (named pAH25-1), an ∼60-kb plasmid (named pAH62-1), and an ∼60-kb plasmid (named pAH65-1) (Fig. S1).

**TABLE 1:** Features of *mcr-1*-positive APEC strains

Strain	Yr	Serotype	MLST	Plasmid	Size (bp)	Plasmid type^*a*^	MDR gene(s)	Accession no.
AH25	2017	O125ab:H28	ST156	pAH25-1	64,941	IncI2	*bla*_CTX-M-55_, *mcr-1*	CP055257
				pAH25-2	116,030	p0111	*strA*, *strB*, *fosA*, *rmtB*, *erm(B)*, *sul1*, *sul2*, *aadA2*, *aadA5*, *floR*, *dfrA17*, *mph*(A), *qepA tet*(A), *dfrA12*, *catA1*, *aph(3′)-Ic*, *aph(4)-Ia*, *aac(3)-Iva*, *bla*_TEM-1B_	CP055258

AH62	2018	O15:H40	ST1788	pAH62-1	60,960	IncI2	*mcr-1*	CP055260
				pAH62-2	84,033	IncI1-I	*strA*, *strB*, *aac(3)-IId*, *sul1*, *sul2*, *mph*(A), *aadA5*, *dfrA17*, *tet*(A), *floR*, *bla*_TEM-1A_	CP055261
				pAH62-3	136,694	IncFIB, IncFIC		CP055262

AH65	2018	O15:H40	ST1788	pAH65-1	60,960	IncI2	*mcr-1*	CP058303
				pAH65-2	84,033	IncI1-I	*strA, strB, aac(3)-IId, sul1, sul2, mph*(A), *aadA5, dfrA17, tet*(A), *floR, bla*_TEM-1A_	CP058304
				pAH65-3	139,513	IncFIB, IncFIC		CP058305

a Plasmid type was determined with PlasmidFinder.

10.1128/mSphere.01135-20.1 FIG S1 S1-PFGE patterns for the three APEC strains and Southern blot analysis for the *mcr-1* gene. Lanes: marker, *Salmonella* serotype Braenderup strain H9812 as the size standard; 1 to 3, PFGE results for S1-digested plasmid DNA of strains AH25, AH62, and AH65; 4 to 6, Southern blotting analysis with the probe specific to *mcr-1*. Download FIG S1, TIF file, 1.6 MB. Copyright © 2021 Yin et al. 2021 Yin et al. https://creativecommons.org/licenses/by/4.0/ This content is distributed under the terms of the Creative Commons Attribution 4.0 International license.

### WGS analysis.

Multiple resistance genes were identified in the three isolates. AH25 carried the *bla*_CTX-M-55_ and *bla*_TEM-1B_ genes, while AH62 and AH65 produced extended-spectrum beta-lactamase (ESBL) with the *bla*_TEM-1A_ gene (Table 1). In addition to the aforementioned MDR genes, the strains were also found to harbor multiple resistance elements, including but not limited to *aadA5*, *floR*, *fosA*, *strA*, *strB*, *sul1*, *sul2*, and *mph*(A), which were consistent with the antimicrobial-resistant phenotypes in the study (Table 1).

**FIG 2:**
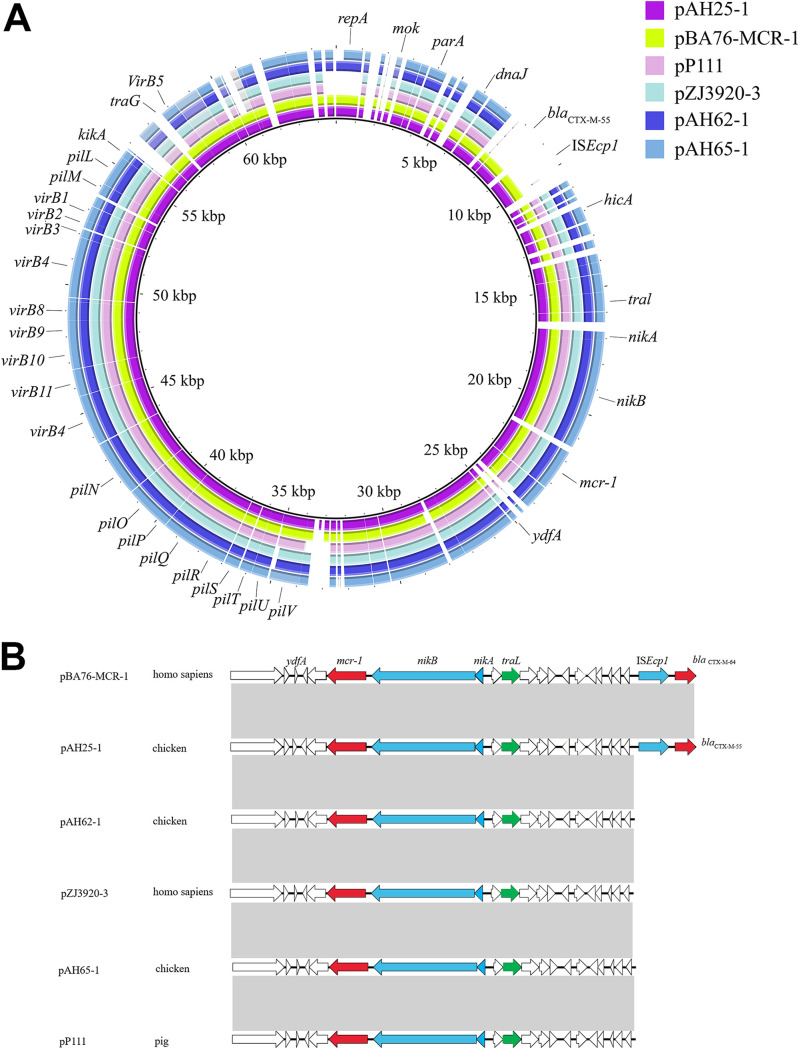
Sequence alignment analysis of IncI2 *mcr-1*-harboring plasmids. (A) Sequence comparison of *mcr-1*-harboring plasmids. (B) Comparative schematic representation of the flanking regions of the *mcr-1* genes in IncI2 plasmids. Areas shaded in gray indicate homologies in the corresponding genetic environment on each plasmid. The open reading frames are shown as boxes or arrows. Antibiotic resistance-encoding genes are indicated with red boxes/arrows. Blue boxes/arrows denote transposon- and integron-associated genes. White boxes/arrows indicate hypothetical proteins or mobile element proteins.

The results of WGS revealed that the three *mcr-1*-harboring plasmids (pAH25-1, pAH62-1, and pAH65-1) belonged to the IncI2 type (Table 1). A BLAST search revealed that the three *mcr-1*-harboring plasmids were highly similar to pBA76-MCR-1 (accession no. KX013540) (100% coverage and 99.56% identity), pZJ3920-3 (accession no. CP020548) (100% coverage and 99.97% identity), and pP111 (accession no. KY120365) (100% coverage and 99.99% identity). The genetic environments of the *mcr-1* gene in the three plasmids and the data available in GenBank were almost the same (Fig. 2A; Fig. S2). The WGS data on the genetic context of the three plasmids in this study revealed a typical plasmid backbone responsible for plasmid replication, maintenance, and transfer (Fig. 2B). Upstream structures were identical to those seen in pAH25-1, pAH62-1, and pAH65-1. In the three plasmids, the *mcr-1*–*pap* cassette was inserted in the conserved cassette position, that is, downstream of the *nikB* locus, and pAH25-1 also harbored an *ISEcp1*-driven *bla*_CTX-M-55_ gene inserted independently of *mcr-1* at the plasmid scaffold (Fig. 2B).

10.1128/mSphere.01135-20.2 FIG S2 Structural features of the *mcr-1*-harboring IncI2 plasmids in this study in comparison with the reference plasmids pBA76-MCR-1, pZJ3920-3, and pP111. Download FIG S2, TIF file, 2.6 MB. Copyright © 2021 Yin et al. 2021 Yin et al. https://creativecommons.org/licenses/by/4.0/ This content is distributed under the terms of the Creative Commons Attribution 4.0 International license.

## DISCUSSION

APEC strains are a versatile group of bacteria revealing a complex phylogeny and considerable genomic plasticity. APEC can cause various disease syndromes, such as pericarditis, perihepatitis, peritonitis, and yolk sac infections in poultry (11). Certain subpopulations of APEC have been suggested to be zoonotic agents (12, 13), which are of great concern because they also have been associated with multidrug-resistant clonal lineages.

All the *mcr-1*-positive transconjugants in this study belonged to IncI2, consistent with the results of previous studies showing that the *mcr-1*-carrying IncI2 plasmid had the advantages of suitability and was more beneficial for the isolation of the host E. coli than either IncHI2 or IncX4 plasmid (14). Since the first report of a plasmid-borne *mcr-1* gene, located on the IncI2 plasmid pHNSHP45, the IncI2 plasmid type has been widely reported as the main vector for *mcr-1* gene transmission worldwide (1). At the same time, the IncI2 plasmid type was also an important carrier of the *bla*_CTX-M_ gene (15). Studies have found that IncI2 plasmids carried the *bla*_CTX-M-55_ gene in E. coli in Chinese pets and food animals. Liu et al. found that IncI2 plasmids harbored the *bla*_CTX-M-64_ gene, which was widely distributed among members of the *Enterobacteriaceae* in different animals and different regions in China (16). Ho et al. reported that IncI2 plasmids in E. coli from pigs and chickens in China carried the *bla*_CTX-M-64_ and *bla*_CTX-M-132_ genes (17). All these findings indicated that the IncI2 plasmid type in different *Enterobacteriaceae* species spread *mcr-1* in food and animals around the world and then to clinical isolates producing carbapenemase.

The existing research results suggest that farms may be the source of the spread of *mcr-1*. This is because the isolation rate of *mcr-1*-positive strains in farms is relatively high, while the isolation rate in hospital-infected patients is relatively low (1). Moreover, farms are closely related to human life, and foodborne bacteria can infect humans through the food chain (18, 19). The *mcr-1*-positive strains are predominantly *Enterobacteriaceae*. On the one hand, bacteria can spread by contaminating animal-derived food; on the other hand, animal excrement returned to the field after being composted may also contaminate crops such as vegetables and wheat (20), thereby threatening public safety.

Fortunately, the use of colistin in animal feed was banned by the Ministry of Agriculture and Rural Affairs of the People's Republic of China (MARA, PRC) (announcement no. 2428; http://www.moa.gov.cn/) in November 2016. The implementation of this policy reduced the selection pressure of drugs on the intestinal flora. In the absence of selective pressure of colistin, the detection rate of the *mcr-1* gene might gradually decrease, but plasmids carrying the *mcr-1* gene also often carry the *bla*_CTX-M_ gene (21). Under the action of amoxicillin, cephalosporin, and other drugs, plasmids carrying both the *bla*_CTX-M_ and *mcr-1* genes or only the *bla*_CTX-M_ gene still persist, which might be the reason why *mcr-1* remained detectable but with a decreased detection rate.

In this study, pAH25-1 was closely aligned with IncI2 plasmid pBA76-MCR-1 from E. coli in humans in the Arabian Peninsula (22). pAH62-1 was closely aligned with IncI2 plasmid pZJ3920-3 from E. coli in humans in Hangzhou, China, and pAH65-1 was closely aligned with IncI2 plasmid pP111 from Salmonella enterica serovar Typhimurium in pigs in Taiwan (23). This phenomenon emphasized that IncI2 plasmid posed a risk for the exchange of genetic material between different niches. Additionally, these *mcr-1*-harboring plasmids lacked the IS*Apl1* element, a key element that mediated the translocation of *mcr-1* into various plasmid backbones and chromosomes (24, 25). It was speculated that the spread of *mcr-1* genes might be dependent on the diffusion of promiscuous plasmids, rather than on the clonal expansion of *mcr-1*-bearing bacteria.

The AH-25 strain belonged to ST156, which is distributed in humans and animals, and was related to human E. coli urinary tract and blood infection isolates (5, 26, 27). It is worth noting that isolates AH-62 and AH-65 both belonged to ST1788, indicating the presence of a clonal kinship. At the same time, the two isolates contained three plasmids of the same type and virulence, but the isolation sites were different, suggesting a trend of potential clonal outbreaks.

In conclusion, E. coli has a wide range of hosts, being isolated from healthy and diseased animals and humans. This study showed that APEC is an important drug resistance gene storehouse, and the prevalence of *mcr-1*-positive APEC posed a potential risk to public health. The surveillance of resistance in the poultry system should be reinforced to curb the transmission or persistence of MDR bacteria due to the wide spread of *mcr-1* around the world; in particular, E. coli easily harbored the plasmid that carried *mcr-1*.

## MATERIALS AND METHODS

### Bacterial collection and detection of resistance genes.

A total of 72 APEC strains were collected from the livers or hearts of diseased chickens in the four urban areas of Anhui Province (Hefei, Anqing, Chuzhou, and Fuyang) from March 2017 to December 2018. The diseased chickens from these farms where the disease occurred were sent to the veterinary hospital of Anhui Agricultural University for testing. The strains were cultured in Luria-Bertani medium at 37°C. The existence of the *mcr-1* gene was determined by PCR amplification (1). MICs of colistin were determined separately using a broth dilution method. An MIC of >2 μg/ml was considered resistant to colistin according to the European Committee on Antimicrobial Susceptibility Testing. In addition, all isolates were tested for their susceptibility to a panel of antimicrobial compounds by the broth dilution method, as described in previous studies (28, 29).

### S1 nuclease–pulsed-field gel electrophoresis, Southern blot analysis, and conjugation assay.

An S1 nuclease–pulsed-field gel electrophoresis (S1-PFGE) assay and Southern blot hybridization were performed to determine the location of the *mcr-1* gene. Genomic DNAs from strains AH25, AH62, and AH65 were digested with the S1 endonuclease (TaKaRa, Dalian, China). DNA fragments were separated by PFGE through a CHEF-DR III system (Bio-Rad, California, USA) with Salmonella enterica serotype Braenderup strain H9812 as a reference size standard. The plasmid DNA was transferred to a positively charged nylon membrane (Solabio, Beijing, China) and hybridized with the digoxigenin-labeled specific probe to *mcr-1*. The *mcr-1* gene conjugation experiments were performed by filter mating using AH25, AH62, and AH65 as the donor and a standard E. coli J53 strain (sodium azide resistant) as the recipient. Cultures of donor and recipient cells in the logarithmic phase were mixed at a ratio of 4:1 and incubated on a brain heart infusion (BHI) agar plate overnight at 37°C without shaking. The transconjugants were selected on BHI plates containing 4 μg/ml colistin and 200 μg/ml sodium azide. Antimicrobial susceptibility testing and PCR were subsequently performed to confirm the transconjugants.

### WGS and bioinformatics analysis.

The genomic DNA of the three isolates was extracted using a High Pure PCR template preparation kit (Roche, Basel, Switzerland). The genomic DNA was subjected to high-throughput sequencing using both Illumina MiSeq and Oxford Nanopore MinION platforms. The *de novo* hybrid assembly of Illumina short reads and MinION long reads was performed using Unicycler version 0.4.8 as the reported method (30). Genome annotation was done by using RAST (31). Acquired antibiotic resistance genes were identified using ResFinder. The sequence type was determined through the multilocus sequence typing (MLST) web server (32). Standard methods were used to annotate the serotype based on the WGS data by CGE platforms. Plasmid replicon types were identified using PlasmidFinder v2.0. Plasmid replicon types and the sequence types of IncF plasmids were identified using PlasmidFinder and pMLST (33). Sequence comparisons and map generation were performed using BLAST and visualized using Easyfig version 2.1 (34).

### Data availability.

The complete sequences of the chromosomes of strains AH25, AH62, and AH65 and plasmids pAH25-1, pAH25-2, pAH62-1, pAH62-2, pAH62-3, pAH65-1, pAH65-2, and pAH65-3 have been deposited in GenBank under the accession numbers CP055256, CP055259, CP058302, CP055257, CP055258, CP055260, CP055261, CP055262, CP058303, CP058304, and CP058305, respectively.

## References

[1] Liu Y-Y, Wang Y, Walsh TR, Yi L-X, Zhang R, Spencer J, Doi Y, Tian G, Dong B, Huang X, Yu L-F, Gu D, Ren H, Chen X, Lv L, He D, Zhou H, Liang Z, Liu J-H, Shen J. 2016. Emergence of plasmid-mediated colistin resistance mechanism *MCR-1* in animals and human beings in China: a microbiological and molecular biological study. Lancet Infect Dis 16:161–168. doi:10.1016/S1473-3099(15)00424-7. 26603172

[2] Shen Z, Wang Y, Shen Y, Shen J, Wu C. 2016. Early emergence of *mcr-1* in Escherichia coli from food-producing animals. Lancet Infect Dis 16:293. doi:10.1016/S1473-3099(16)00061-X. 26973308

[3] Rhouma M, Beaudry F, Theriault W, Letellier A. 2016. Colistin in pig production: chemistry, mechanism of antibacterial action, microbial resistance emergence, and One Health perspectives. Front Microbiol 7:1789. doi:10.3389/fmicb.2016.01789. 27891118 PMC5104958

[4] Gao R, Hu Y, Li Z, Sun J, Wang Q, Lin J, Ye H, Liu F, Srinivas S, Li D, Zhu B, Liu YH, Tian GB, Feng Y. 2016. Dissemination and mechanism for the *MCR-1* colistin resistance. PLoS Pathog 12:e1005957. doi:10.1371/journal.ppat.1005957. 27893854 PMC5125707

[5] Wang Y, Tian G-B, Zhang R, Shen Y, Tyrrell JM, Huang X, Zhou H, Lei L, Li H-Y, Doi Y, Fang Y, Ren H, Zhong L-L, Shen Z, Zeng K-J, Wang S, Liu J-H, Wu C, Walsh TR, Shen J. 2017. Prevalence, risk factors, outcomes, and molecular epidemiology of *mcr-1*-positive *Enterobacteriaceae* in patients and healthy adults from China: an epidemiological and clinical study. Lancet Infect Dis 17:390–399. doi:10.1016/S1473-3099(16)30527-8. 28139431

[6] Ellem JA, Ginn AN, Chen SC, Ferguson J, Partridge SR, Iredell JR. 2017. Locally acquired *mcr-1* in *Escherichia coli*, Australia, 2011 and 2013. Emerg Infect Dis 23:1160–1163. doi:10.3201/eid2307.161638. 28628439 PMC5512495

[7] Perrin-Guyomard A, Bruneau M, Houee P, Deleurme K, Legrandois P, Poirier C, Soumet C, Sanders P. 2016. Prevalence of *mcr-1* in commensal Escherichia coli from French livestock, 2007 to 2014. Euro Surveill 21:30135. doi:10.2807/1560-7917.ES.2016.21.6.30135. 26898350

[8] Monte DF, Mem A, Fernandes MR, Cerdeira L, Esposito F, Galvao JA, Franco B, Lincopan N, Landgraf M. 2017. Chicken meat as a reservoir of colistin-resistant *Escherichia coli* strains carrying *mcr-1* genes in South America. Antimicrob Agents Chemother 61:e02718-16. doi:10.1128/AAC.02718-16. 28193665 PMC5404526

[9] Wang Y, Zhang R, Li J, Wu Z, Yin W, Schwarz S, Tyrrell JM, Zheng Y, Wang S, Shen Z, Liu Z, Liu J, Lei L, Li M, Zhang Q, Wu C, Zhang Q, Wu Y, Walsh TR, Shen J. 2017. Comprehensive resistome analysis reveals the prevalence of *NDM* and *MCR-1* in Chinese poultry production. Nat Microbiol 2:16260. doi:10.1038/nmicrobiol.2016.260. 28165472

[10] Schrauwen EJA, Huizinga P, van Spreuwel N, Verhulst C, Kluytmans-van den Bergh MFQ, Kluytmans J. 2017. High prevalence of the *mcr-1* gene in retail chicken meat in the Netherlands in 2015. Antimicrob Resist Infect Control 6:83. doi:10.1186/s13756-017-0242-8. 28828173 PMC5563067

[11] Ewers C, Li G, Wilking H, Kiessling S, Alt K, Antao EM, Laturnus C, Diehl I, Glodde S, Homeier T, Bohnke U, Steinruck H, Philipp HC, Wieler LH. 2007. Avian pathogenic, uropathogenic, and newborn meningitis-causing *Escherichia coli*: how closely related are they? Int J Med Microbiol 297:163–176. doi:10.1016/j.ijmm.2007.01.003. 17374506

[12] Mellata M. 2013. Human and avian extraintestinal pathogenic *Escherichia coli*: infections, zoonotic risks, and antibiotic resistance trends. Foodborne Pathog Dis 10:916–932. doi:10.1089/fpd.2013.1533. 23962019 PMC3865812

[13] Nandanwar N, Janssen T, Kuhl M, Ahmed N, Ewers C, Wieler LH. 2014. Extraintestinal pathogenic *Escherichia coli* (ExPEC) of human and avian origin belonging to sequence type complex 95 (STC95) portray indistinguishable virulence features. Int J Med Microbiol 304:835–842. doi:10.1016/j.ijmm.2014.06.009. 25037925

[14] Wu R, Yi LX, Yu LF, Wang J, Liu Y, Chen X, Lv L, Yang J, Liu JH. 2018. Fitness advantage of *mcr-1*-bearing IncI2 and IncX4 plasmids in vitro. Front Microbiol 9:331. doi:10.3389/fmicb.2018.00331. 29535696 PMC5835064

[15] Wong MH, Liu L, Yan M, Chan EW, Chen S. 2015. Dissemination of IncI2 plasmids that harbor the *bla*CTX-M element among clinical *Salmonella* isolates. Antimicrob Agents Chemother 59:5026–5028. doi:10.1128/AAC.00775-15. 26014934 PMC4505288

[16] Liu L, He D, Lv L, Liu W, Chen X, Zeng Z, Partridge SR, Liu JH. 2015. *bla*CTX-M-1/9/1 hybrid genes may have been generated from blaCTX-M-15 on an IncI2 plasmid. Antimicrob Agents Chemother 59:4464–4470. doi:10.1128/AAC.00501-15. 25987615 PMC4505238

[17] Ho PL, Liu MC, Lo WU, Lai EL, Lau TC, Law OK, Chow KH. 2015. Prevalence and characterization of hybrid *bla*CTX-M among *Escherichia coli* isolates from livestock and other animals. Diagn Microbiol Infect Dis 82:148–153. doi:10.1016/j.diagmicrobio.2015.02.010. 25861872

[18] Ma L, Wang Y, Shen J, Zhang Q, Wu C. 2014. Tracking Campylobacter contamination along a broiler chicken production chain from the farm level to retail in China. Int J Food Microbiol 181:77–84. doi:10.1016/j.ijfoodmicro.2014.04.023. 24831929

[19] Rodriguez C, Taminiau B, Avesani V, Van Broeck J, Delmee M, Daube G. 2014. Multilocus sequence typing analysis and antibiotic resistance of Clostridium difficile strains isolated from retail meat and humans in Belgium. Food Microbiol 42:166–171. doi:10.1016/j.fm.2014.03.021. 24929733

[20] Luo J, Yao X, Lv L, Doi Y, Huang X, Huang S, Liu J-H. 2017. Emergence of *mcr-1* in *Raoultella ornithinolytica* and *Escherichia coli* isolates from retail vegetables in China. Antimicrob Agents Chemother 61:e01139-17. doi:10.1128/AAC.01139-17. 28739785 PMC5610531

[21] Faccone D, Moredo FA, Giacoboni GI, Albornoz E, Alarcon L, Nievas VF, Corso A. 2019. Multidrug-resistant Escherichia coli harbouring *mcr-1* and *bla*CTX-M genes isolated from swine in Argentina. J Glob Antimicrob Resist 18:160–162. doi:10.1016/j.jgar.2019.03.011. 30926466

[22] Sonnevend A, Ghazawi A, Alqahtani M, Shibl A, Jamal W, Hashmey R, Pal T. 2016. Plasmid-mediated colistin resistance in *Escherichia coli* from the Arabian Peninsula. Int J Infect Dis 50:85–90. doi:10.1016/j.ijid.2016.07.007. 27566913

[23] Liu BT, Song FJ. 2019. Emergence of two *Escherichia coli* strains co-harboring *mcr-1* and *bla*NDM in fresh vegetables from China. Infect Drug Resist 12:2627–2635. doi:10.2147/IDR.S211746. 31692544 PMC6711560

[24] Li R, Yu H, Xie M, Chen K, Dong N, Lin D, Chan EW, Chen S. 2018. Genetic basis of chromosomally-encoded *mcr-1* gene. Int J Antimicrob Agents 51:578–585. doi:10.1016/j.ijantimicag.2017.11.015. 29197647

[25] Tegetmeyer HE, Jones SC, Langford PR, Baltes N. 2008. ISApl1, a novel insertion element of Actinobacillus pleuropneumoniae, prevents ApxIV-based serological detection of serotype 7 strain AP76. Vet Microbiol 128:342–353. doi:10.1016/j.vetmic.2007.10.025. 18065168

[26] Kong LH, Lei CW, Ma SZ, Jiang W, Liu BH, Wang YX, Guan R, Men S, Yuan QW, Cheng GY, Zhou WC, Wang HN. 2017. Various sequence types of *Escherichia coli* isolates coharboring *bla*(NDM-5) and *mcr-1* genes from a commercial swine farm in China. Antimicrob Agents Chemother 61:e02167-16. doi:10.1128/AAC.02167-16. 27993847 PMC5328568

[27] Yang RS, Feng Y, Lv XY, Duan JH, Chen J, Fang LX, Xia J, Liao XP, Sun J, Liu YH. 2016. Emergence of *NDM-5*- and *MCR-1*-producing *Escherichia coli* clones ST648 and ST156 from a single Muscovy duck (Cairina moschata). Antimicrob Agents Chemother 60:6899–6902. doi:10.1128/AAC.01365-16. 27550364 PMC5075103

[28] Bell S, Gatus B, Pham J, Rafferty D. 1999. Antibiotic susceptibility testing by the CDS method. A concise laboratory manual. South Eastern Area Laboratory Services, The CDS Reference Laboratory, Kogarah, Australia.

[29] Clinical and Laboratory Standards Institute. 2018. Performance standards for antimicrobial susceptibility testing: 20th informational supplement. CLSI document M100-S28. Clinical and Laboratory Standards Institute, Wayne, PA.

[30] Wick RR, Judd LM, Gorrie CL, Holt KE. 2017. Unicycler: resolving bacterial genome assemblies from short and long sequencing reads. PLoS Comput Biol 13:e1005595. doi:10.1371/journal.pcbi.1005595. 28594827 PMC5481147

[31] Aziz RK, Bartels D, Best AA, DeJongh M, Disz T, Edwards RA, Formsma K, Gerdes S, Glass EM, Kubal M, Meyer F, Olsen GJ, Olson R, Osterman AL, Overbeek RA, McNeil LK, Paarmann D, Paczian T, Parrello B, Pusch GD, Reich C, Stevens R, Vassieva O, Vonstein V, Wilke A, Zagnitko O. 2008. The RAST server: rapid annotations using subsystems technology. BMC Genomics 9:75. doi:10.1186/1471-2164-9-75. 18261238 PMC2265698

[32] Larsen MV, Cosentino S, Rasmussen S, Friis C, Hasman H, Marvig RL, Jelsbak L, Sicheritz-Pontén T, Ussery DW, Aarestrup FM, Lund O. 2012. Multilocus sequence typing of total-genome-sequenced bacteria. J Clin Microbiol 50:1355–1361. doi:10.1128/JCM.06094-11. 22238442 PMC3318499

[33] Carattoli A, Zankari E, García-Fernández A, Voldby Larsen M, Lund O, Villa L, Møller Aarestrup F, Hasman H. 2014. In silico detection and typing of plasmids using PlasmidFinder and plasmid multilocus sequence typing. Antimicrob Agents Chemother 58:3895–3903. doi:10.1128/AAC.02412-14. 24777092 PMC4068535

[34] Sullivan MJ, Petty NK, Beatson SA. 2011. Easyfig: a genome comparison visualizer. Bioinformatics 27:1009–1010. doi:10.1093/bioinformatics/btr039. 21278367 PMC3065679

