# Supplementary material for: Complete Genetic Analysis of Plasmids Carrying mcr-1 and Other Resistance Genes in Avian Pathogenic Escherichia coli Isolates from Diseased Chickens in Anhui Province in China
Source: mSphere. 2021 Apr 14;6(2):e01135-20. doi: 10.1128/mSphere.01135-20 (PMC8546713; doi:10.1128/mSphere.01135-20)
Supplement: TABLE S1 [file msphere.01135-20-st001.docx]

**TABLE S1.** Antibiotic susceptibilities of the three APEC strains and their transconjugants with plasmids either harboring *mcr-1*

| Isolates | Antimicrobial | | | | | | | |
| --- | --- | --- | --- | --- | --- | --- | --- | --- |
|  | AMP | AMX | CQM | TET | KAN | FFC | FOS | COL |
| AH25 | >64 | >64 | >64 | >32 | >32 | >128 | >128 | 8 |
| AH25COL | >32 | >64 | >64 | ≤4 | ≤2 | 4 | 4 | 8 |
| AH62 | >32 | >64 | ≤0.25 | >32 | >32 | >64 | 4 | 8 |
| AH62COL | 8 | ≤1 | ≤0.25 | ≤4 | ≤2 | 4 | 4 | 8 |
| AH65 | >32 | >64 | ≤0.25 | >32 | >32 | >64 | 4 | 8 |
| AH65COL | 8 | ≤1 | ≤0.25 | ≤4 | ≤2 | 4 | 4 | 8 |
| J53 | 8 | ≤1 | ≤0.25 | ≤4 | ≤2 | 4 | 4 | 0.5 |

AMP: Ampicillin; AMX: amoxicillin; CQM: cefquinome; TET: Tetracycline; KAN: Kanamycin; FFC: florfenicol; FOS: Fosfomycin; COL: colistin.
