# Supplementary figures and images for: Complete Genetic Analysis of Plasmids Carrying mcr-1 and Other Resistance Genes in Avian Pathogenic Escherichia coli Isolates from Diseased Chickens in Anhui Province in China
Source: mSphere. 2021 Apr 14;6(2):e01135-20. doi: 10.1128/mSphere.01135-20 (PMC8546713; doi:10.1128/mSphere.01135-20)

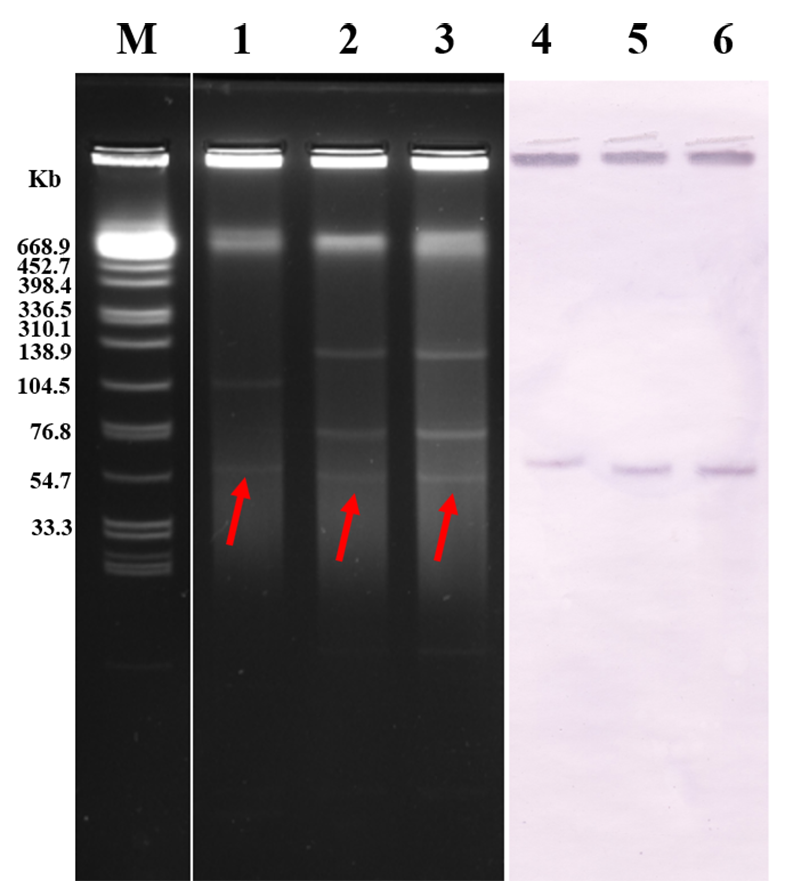

Supplement: FIG S1 [file msphere.01135-20-sf001.tif]

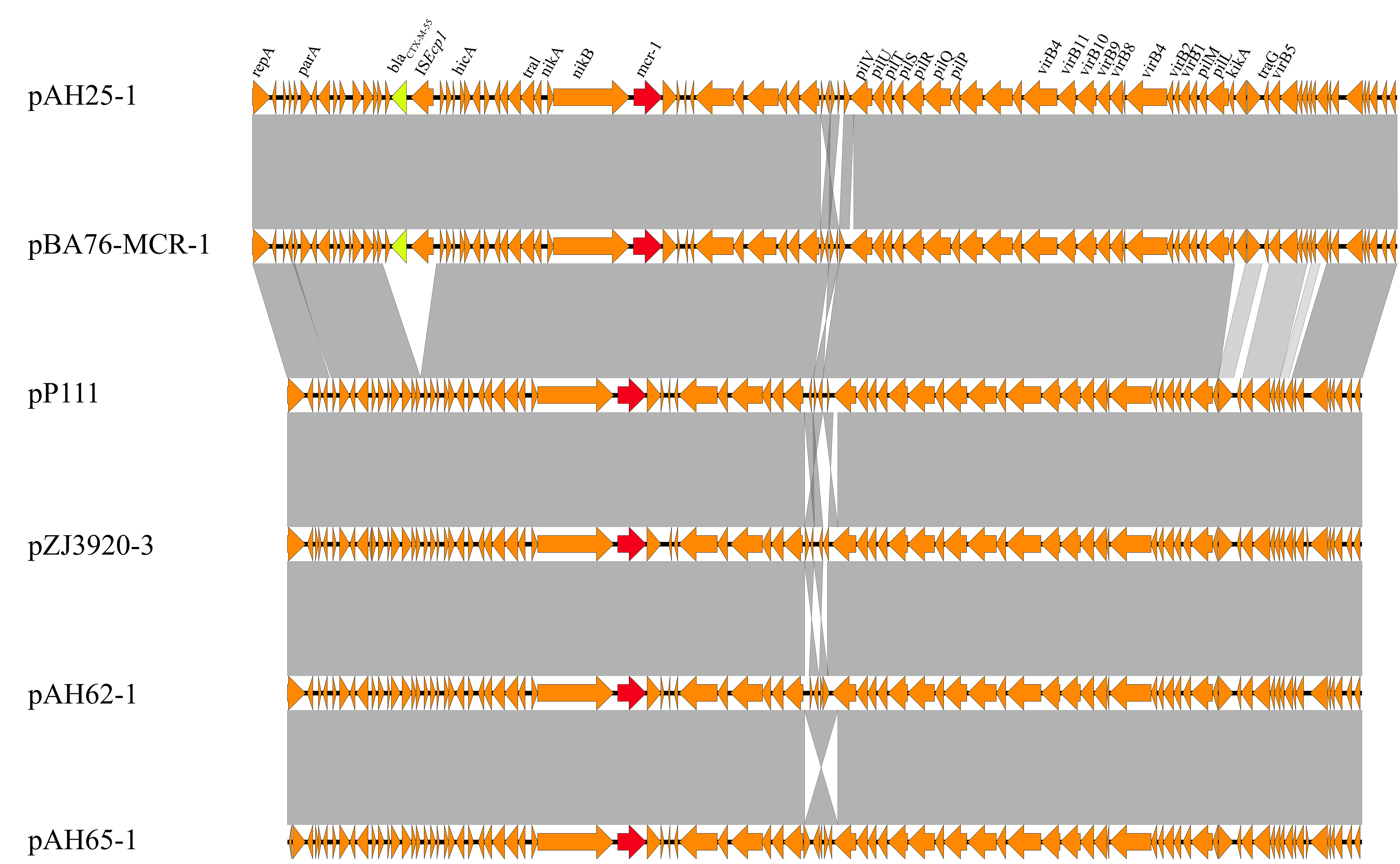

Supplement: FIG S2 [file msphere.01135-20-sf002.tif]
